# Molecular surveillance of multidrug-resistant Gram-negative bacteria in Ukrainian patients, Germany, March to June 2022

**DOI:** 10.2807/1560-7917.ES.2023.28.1.2200850

**Published:** 2023-01-05

**Authors:** Tilman Schultze, Michael Hogardt, Erwin Sanabria Velázquez, Daniel Hack, Silke Besier, Thomas A Wichelhaus, Ulrich Rochwalsky, Volkhard AJ Kempf, Claudia Reinheimer

**Affiliations:** 1 Institute for Medical Microbiology and Infection Control, University Hospital Frankfurt, Frankfurt am Main, Germany; 2 University Centre of Infectious Diseases, University Hospital Frankfurt, Frankfurt am Main, Germany; 3 University Centre of Competence for Infection Control of the State of Hesse, Frankfurt am Main, Frankfurt Main, Germany; 4 Department of Paediatrics, University Hospital Frankfurt, Frankfurt am Main, Germany

**Keywords:** Ukraine, refugees, multidrug-resistant gram-negative bacteria, molecular surveillance

## Abstract

**Background:**

Since the beginning of the war in Ukraine in February 2022, Ukrainians have been seeking shelter in other European countries.

**Aim:**

We aimed to investigate the prevalence and the molecular epidemiology of multidrug-resistant Gram-negative (MDRGN) bacteria and meticillin-resistant *Staphylococcus aureus* (MRSA) in Ukrainian patients at admittance to the University Hospital Frankfurt, Germany.

**Methods:**

We performed screening and observational analysis of all patients from March until June 2022. Genomes of MDRGN isolates were analysed for antimicrobial resistance, virulence genes and phylogenetic relatedness.

**Results:**

We included 103 patients (median age: 39 ± 23.7 years), 57 of whom were female (55.3%; 95% confidence interval (CI): 45.2–5.1). Patients were most frequently admitted to the Department of Paediatrics (29/103; 28.2%; 95% CI: 19.7–37.9). We found 34 MDRGN isolates in 17 of 103 patients (16.5%; 95% CI: 9.9–25.1). Ten patients carried 21 carbapenem-resistant (CR) bacteria, five of them more than one CR isolate. Four of six patients with war-related injuries carried eight CR isolates. In six of 10 patients, CR isolates caused infections. Genomic characterisation revealed that the CR isolates harboured at least one carbapenemase gene, *bla*_NDM-1_ being the most frequent (n = 10). Core genome and plasmid analysis revealed no epidemiological connection between most of these isolates. Hypervirulence marker genes were found in five of six *Klebsiella pneumoniae* CR isolates. No MRSA was found.

**Conclusion:**

Hospitals should consider infection control strategies to cover the elevated prevalence of MDRGN bacteria in Ukrainian patients with war-related injuries and/or hospital pre-treatment and to prevent the spread of hypervirulent CR isolates.

**Key public health message**

**What did you want to address in this study?**

What is the prevalence of multidrug- and carbapenem-resistant Gram-negative bacteria in patients arriving from Ukraine? Are these bacterial isolates related and how dangerous are they for humans?

**What have we learnt from this study?**

Carbapenem-resistant bacteria were frequently found among Ukrainian patients, especially in children, patients with war-related injuries or after medical pre-treatment in Ukraine. Genetic characterisation revealed nine carbapenemase genes, with NDM-1 detected most frequently. We found hypervirulence marker genes in five of six carbapenem-resistant *Klebsiella pneumoniae* isolates. In core genome analysis and plasmid typing, we saw no epidemiological link between the infections.

**What are the implications of your findings for public health?**

The high prevalence of multidrug- and carbapenem-resistant bacteria and the occurrence of hypervirulent carbapenem-resistant *K. pneumonia *strains in Ukrainian patients may contribute to further spread of these pathogens in Europe. In hospitals, stringent infection control strategies are needed to avoid transmission of these bacteria reliably.

## Introduction

Since the beginning of the recent war in Ukraine in February 2022 [1], around 1,010,000 Ukrainian refugees have been registered in Germany by end of October 2022 [2,3]. Since exposure to war and traumatic or uprooting experiences are known to impact both mental and somatic health [4-8], refugees are expected to seek medical advice in the countries giving shelter. 

Data from the European Centre for Disease Prevention and Control (ECDC) indicate a high prevalence of invasive multidrug-resistant Gram-negative (MDRGN) bacteria in Ukraine in the year 2020, when almost 77% of *Acinetobacter baumannii* and 84% of *Klebsiella pneumoniae* were found to be carbapenem-resistant (CR) [9]. In addition, multidrug-resistant bacteria in patients with war-related injuries, e.g. CR* A. baumannii*, are a well-described issue of concern [10,11]. Patients arriving from Ukraine and in particular patients suffering from war-related injuries are therefore expected to be at elevated risk of carrying MDRGN pathogens. Knowledge on the epidemiology of MDRGN bacteria in these patients is lacking, although such data are crucial for infection prevention and control. 

This study addresses the prevalence and molecular epidemiology of MDRGN bacteria in Ukrainian patients admitted to the University Hospital Frankfurt am Main, Germany (UHF) since the beginning of the war in Ukraine in February 2022. Our study contributes infection control management of patients transferred from countries affected by war (here: Ukraine) to guarantee best medical care for each individual patient.

## Methods

### Setting

All patients who reported leaving the Ukraine were screened on the day of admittance to the UKF from March until June 2022. The patients’ details (as far as known) were taken from patient data files. The patients’ history of leaving Ukraine was also recorded in the patient data files by the physicians on the date of admission. If this entry was missing, the respective patient was excluded. The patients’ age is given in accordance with age groups introduced by the World Health Organization (WHO) in 2001 [12].

### Screening procedures at the University Hospital Frankfurt am Main, Germany

German infection protection law (*Infektionsschutzgesetz* [13]) mandatorily requires a documented infection control protocol to prevent nosocomial infections. At the UHF, all patients admitted from hospitals or from countries with high MDRGN prevalence are pre-emptively isolated and screened for MDRGN bacteria and meticillin-resistant *Staphylococcus aureus* (MRSA) on the day of admission. In Germany, screening for vancomycin-resistant *Enterococcus* spp. (VRE) is not mandatory except for patients in high-risk settings and therefore, routine VRE screening was not performed. Further pathogens like *Stenotrophomonas maltophilia* are not part of any routine infection prevention screening recommendations in Germany. The screening procedure also applies to every patient admitted to any intensive care unit. Immediately after negative results are available, the patients are released from isolation. If MDRGN bacteria or MRSA are detected, the patient will usually remain in isolation during their entire stay to prevent pathogen transmission [14,15]. 

### Detection of meticillin-resistant *Staphylococcus aureus*, multidrug-resistant Gram-negative bacteria and molecular resistance analysis

According to existing definitions [14], MDRGN bacteria were defined as *Enterobacterales *with extended spectrum beta-lactamase (ESBL) phenotype and additional resistance against fluoroquinolones (FQ) and/or carbapenems as well as *Enterobacterales*, *Acinetobacter baumannii* and *Pseudomonas aeruginosa* resistant to piperacillin, any third or fourth generation cephalosporin and FQ and/or with carbapenem resistance.

All laboratory testing was performed under strict quality-controlled DIN ISO 15189:2007 standards (certificate number D–ML–13102–01–00) as previously described [14,15]. For MRSA screening, nasopharyngeal swabs were obtained routinely. For MDRGN screening, rectal swabs were collected, using culture swabs with Amies collection and transport medium (Hain Lifescience, Nehren, Germany). If applicable, swabs from wounds or tracheal secretion were also taken. Bacterial cultivation was done on selective Brilliance MRSA Agar (Oxoid, Wesel, Germany) and CHROMagarTM ESBL plates (Mast Diagnostica, Paris, France) without additional enrichment cultures. Species identification was done by matrix-assisted laser desorption ionization-time of flight analysis (MALDI–TOF) and VITEK2 (bioMérieux, Nürtingen, Germany). Antibiotic susceptibility testing of suspected MDRGN isolates was performed according to European Committee on Antimicrobial Susceptibility Testing (EUCAST) guidelines [16] using VITEK 2 and antibiotic gradient tests (bioMérieux). Carbapenemase-encoding genes were detected by PCR and subsequent sequencing of CR *Enterobacterales* including the *bla* genes for carbapenemases NDM, VIM, IMP, OXA–48-like and KPC as well as OXA–23, OXA–24 and OXA–58 for *A. baumannii* [17,18].

### Sequencing and bioinformatics analysis

Whole genome sequencing and sequence analysis was done as previously described [19]. Briefly, DNA was extracted from bacterial cultures using a DNeasy UltraClean 96 Kit (Qiagen, Venlo, Netherlands). Library preparation and sequencing was performed by a commercial service provider (Novogene, Cambridge, United Kingdom) using Illumina chemistry. Sequencing was carried out on a NovaSeq 6000 flow cell using a paired-end sequencing strategy of 2 × 150 bp. Analysis of phylogenetic relatedness was carried out using the pan-genome pipeline Roary [20]. *In silico* screening for antimicrobial resistance genes was performed with ABRicate (accessible at https://github.com/tseemann/abricate) using the Comprehensive Antibiotic Resistance Database (CARD) [21] as reference. We also used ABRicate to screen the genomes of *K. pneumoniae* isolates for hypervirulence genes by searching against gene entries of hypervirulence plasmid pLVPK (NC_005249.1) as the reference. Plasmid-related sequences were analysed with RFplasmid (accessible at http://klif.uu.nl/rfplasmid) and plasmidMLST (accessible at https://pubmlst.org/bacteria/plasmid-mlst). All sequence data generated for this study were deposited in the National Center for Biotechnology Information (NCBI) Sequence Read Archive (https://www.ncbi.nlm.nih.gov/sra) and are available under BioProject accession number PRJNA891092.

### Statistical analysis

Chi-squared test was performed for statistical analysis, p values ≤ 0.05 were considered statistically significant. 95% confidence intervals (CI) for frequencies were calculated based on binomial distribution.

## Results

### Patient characteristics

Within the first 4 months since the beginning of the war in Ukraine, 103 Ukrainian patients were screened for MDRGN bacteria and MRSA on the day of hospital admittance. The median age was 39 ± 23.7 years. Of all 103 patients, 57 (55.3%; 95% CI: 45.2–5.1) were females. Patients were most frequently admitted to the Department of Paediatrics (n=29/103; 28.2%; 95% CI: 19.7–37.9). Additional information is provided in Table 1.

**Table 1:** Characteristics of Ukrainian patients, including hospital departments and microbiological findings, Frankfurt am Main, Germany, March–June 2022 (n = 103)

Characteristic		n	%	95%CI
Patients (n = 103)				
Female		57	55.3	NA
Male		46	44.7	NA
Median age in years (standard deviation)		39 years (23.7)		
Department patients were admitted to				
Paediatric and adolescent medicine		29	28.2	19.7–37.9
Gastroenterology		12	11.7	6.2–19.5
Obstetrics and gynaecology		11	10.7	5.4–18.3
Haematology/oncology		9	8.7	4.1–15.9
General and visceral surgery; Trauma, hand and reconstructive surgery		14 (7 each)	6.8	2.8–13.5
Cardiology		5	4.9	1.6–11.0
Otorhinolaryngology, head and neck surgery; Neurology		8 (4 each)	3.9	1.1–9.6
Vascular and endovascular surgery; Nephrology; Pneumology; Child and adolescent psychiatry, psychosomatics and psychotherapy		8 (2 each)	1.9	0.2–6.8
Other^a^		7	6.8	2.8–13.5
Patients positive for				
MRSA		0	0.0	0.0–3.5
≥ 1 MDRGN isolate		17	16.5	9.9–25.1
≥ 1 CR isolate		10	9.7	4.8–17.1
< 18 years of age among the CR-positive		4	40.0	12.2–73.8
MDRGN isolates in Ukrainian patients (n = 34)				
CR		21	61.8	43.6–77.8
*Escherichia coli*	ESBL, FQ	8	23.5	10.7–41.2
	CR	5	14.7	5.0–31.1
	*C*arbapenemases (n)	NDM-5 (2); NDM-1 (1); OXA-48 (1); KPC-3 + NDM-5 (1)		
*Klebsiella pneumoniae*	ESBL, FQ	2	5.9	0.7–19.7
	CR	6	17.6	6.8–34.5
	Carbapenemases (n)	NDM-1 (2); NDM-1 + OXA-48 (3); NDM-1 + OXA-244 (1)		
*Enterobacter cloacae*	Ceph, FQ	2	5.9	0.7–19.7
*Acinetobacter baumannii*	CR	5	14.7	5.0–31.1
	Carbapenemase (n)	OXA-72 (3); OXA-23 (1); OXA-23 + OXA-72 (1)		
*Proteus mirabilis*	Ceph, FQ	1	2.9	0.0–15.3
	CR	1	2.9	0.0–15.3
	Carbapenemase (n)	NDM-1 (1)		
*Providencia stuartii*	CR	1	2.9	0.0–15.3
	Carbapenemase (n)	NDM-1 (1)		
*Pseudomonas aeruginosa*	Pip, Ceph, FQ, CR	3	8.8	1.9–23.7
	Carbapenemase (n)	NDM-1 (1); IMP-34 (1); GES-1 (1)		

Ceph: resistant to third/fourth generation cephalosporins; CI: confidence interval; CR: resistant to carbapenems; ESBL: extended spectrum beta-lactamase phenotype; FQ: resistant to fluoroquinolones; MDRGN: multidrug-resistant Gram-negative; MRSA: meticillin-resistant *Staphylococcus aureus*; Pip: resistant to piperacillin; NA: not available.

^a^ Departments of urology, rheumatology, infectious diseases, ophthalmology, neurosurgery, psychiatry, psychosomatic medicine and psychotherapy and orthopaedics with n=1 each.

### Carriage of MDRGN bacteria and MRSA in the study patient group

In total, 17 of the 103 patients (16.5%; 95% CI: 9.9–25.1) tested positive for 34 MDRGN isolates. None of these patients were colonised or infected with MRSA. The patients’ median age was 40 ± 23.8 years and six of the 17 were female. Six of the 17 patients had war-related injuries and five were ≤ 18 years of age. Among the MDRGN species, ESBL-producing *Escherichia coli* with additional resistance to fluoroquinolones (8/34 isolates) were most frequent. Several of these isolates belonged to different globally distributed sequence types of high-risk multidrug-resistant clones (ST131, ST410 and ST1193 [22-24]). All isolates carried CTX-M-type ESBL (e.g. *bla*_CTX-M-15_ or *bla*_CTX-M-27_). Of the 34 MDRGN isolates, 29 were available for further molecular characterisation. A comprehensive table of extra antimicrobial resistance genes identified in the genome of isolates of this study is included in the Supplement. No nosocomial transmissions between Ukrainian patients or to any other patients were detected at the UHF.

### Carriage of carbapenem-resistant bacteria in the study patient group

In total, 21 CR isolates were detected in 10 of the 103 patients, resulting in an overall prevalence of 9.7% (95% CI: 4.8–17.1; Table 1). The patients’ median age was 36 ± 23.9 years. Among the 10 positive for CR bacteria, six patients were male, four patients were female. Three of the 10 patients positive for CR bacteria (Table 1) were children in the age group 0–4 years. All 10 reported medical pre-treatment in Ukraine, and four of them had war-related injuries. Five of the 10 patients tested multi-positive for CR bacteria. Among these five, one was positive for four different CR isolates, three were positive for three different CR isolates each and one was positive for two CR isolates (Table 2). In six of 10 patients with CR bacteria, the respective isolates were causing infections (e.g. from surgical sites). All patients positive for CR bacteria reported medical pre-treatment in Ukraine within the previous 12 months (Table 2). 

**Table 2:** Characteristics of Ukrainian patients tested positive for carbapenem-resistant Gram-negative bacteria on the day of admittance, Frankfurt am Main, Germany, March–June 2022 (n = 10)

Patient	Age group (years)^a^	WI	MDRGN+CR bacteria	Carbapenemase(s)	Sequence type	Plasmid profile
A	0–4	No	*Acinetobacter baumannii*	OXA-72	78	NT
B			*Acinetobacter baumannii*	OXA-72	78	NT
C	45–49	Yes	*Acinetobacter baumannii*	OXA-23	2	NT
			*Klebsiella pneumoniae*	NDM-1	395	NT
			*Pseudomonas aeruginosa*	IMP-34	1047	NT
D	50–54	Yes	*Acinetobacter baumannii*	OXA-23 + OXA-72	NT	NT
			*Klebsiella pneumoniae*	NDM-1 + OXA-244	392	NT
			*Pseudomonas aeruginosa*	NDM-1	773	NT
E	15–19	Yes	*Klebsiella pneumoniae*	NDM-1 + OXA-48	23	NT
			*Escherichia coli*	NDM-5	46	IncI1 MLST: ST57
F	50–54	Yes	*Klebsiella pneumoniae*	NDM-1	23	NT
G	0–4	No	*Acinetobacter baumannii*	OXA-72	78	NT
			*Klebsiella pneumoniae*	NDM-1 + OXA-48	147	IncA/C cgPMLST: cgST1.2; IncA/C PMLST: ST1
			*Escherichia coli*	NDM-1	11240	IncA/C cgPMLST: cgST1.2; IncA/C PMLST: ST1; IncI1 MLST: ST264
H	65–69	No	*Escherichia coli*	NDM-5	46	IncI1 MLST: ST57
I	25–29	No	*Escherichia coli*	OXA-48	354	NT
J	40–44	No	*Klebsiella pneumoniae*	NDM-1 + OXA-48	147	NT
			*Escherichia coli*	KPC-3 + NDM-5	361	NT
			*Proteus mirabilis*	NDM-1	NT	NT
			*Pseudomonas aeruginosa*	GES-1	235	NT

CR: carbapenem-resistant; MDRGN: multidrug-resistant Gram-negative; NT: non-typable isolates; WI: war-related injuries.

^a^ Age groups in accordance with the World Health Organization [19].

The largest group of CR species (and the second largest group overall) was formed by *K. pneumoniae* (6/34 isolates; Table 1). Of these, ST23 accounted for two isolates and CC147 accounted for three isolates (two ST147 and one ST392). Both ST23 and ST147 are associated with hypervirulent *K. pneumoniae* (hvKp). While association between ST23 and hypervirulence is known [25], outbreaks of hypervirulent ST147 have emerged recently [26,27]. We provide in Supplementary Table S2 the results of a search for genes of hypervirulence plasmid pLVPK (coding for hypervirulence markers) where five of the six CR *K. pneumoniae* were positive. Of note, the number of pLVPK-associated genes found in the genomes of the isolates varied, indicating the presence of different plasmids in these isolates.

### Patients with war-related injuries

Six of 103 patients (5.8%; 95% CI: 2.2–12.2, all male) had war-related injuries. The median age was 42.5 ± 9.8 years. Furthermore, all six were positive for MDRGN or CR bacteria, with a total of 13 MDRGN and CR isolates. Four of the six patients were positive for between one and three different CR isolates, resulting in nine CR isolates in total (Table 2). In patients without war-related injuries or medical pre-treatment in a Ukrainian hospital prior to admission to the UHF (12/103 patients), prevalence of MDRGN bacteria amounted to 5.5%.

### Core genome analysis

In total, 34 isolates were cultured of which we analysed 29. The remaining five bacterial isolates were not stored in the routine laboratories and therefore not included in this analysis. Core genome analysis of all 29 bacterial isolates revealed a close relation between three pairs indicated by < 20 single nucleotide variances (SNV) and a fourth, more distantly related pair. The closely related pairs were MDRGN *Enterobacter cloacae* and CR *A. baumannii* isolates derived from patients A and B (siblings). Besides, a pair of *E. coli* CR isolates (patients E and H) were identified for which analysis of patient records did not yield any obvious epidemiological linkage (e.g. pre-treatment in the same hospital, transportation by the same medical airplane). The more distantly related pair was formed by *K. pneumoniae* CR isolates (patients E and F) which were separated by about 50 SNV in the core genome (4,174 genes). These two isolates differed also regarding detected carbapenemases (singular NDM-1 versus NDM-1 and OXA-48). A precise analysis of the patient records did not reveal any obvious epidemiological linkage. Plasmid profiling of all 29 isolates revealed identical profiles for two of the isolate pairs which were also found to be closely related based on core genome analysis (MDRGN *E. cloacae* isolates from patients A and B or CR *E. coli *isolates from patient E and H, respectively; see Table 2). In addition, similar plasmid types were found for a *K. pneumoniae* (NDM‑1 and OXA‑48) and an *E. coli* isolate (NDM‑1) derived from the same patient H indicating a potential *in vivo* transfer. A detailed analysis of all plasmid allele types is appended in Supplementary Figure S1; it revealed that four *A. baumannii* isolates carried identical plasmids harbouring OXA-72 hinting for an earlier obtained plasmid transfer.

## Discussion

Our data demonstrate a high prevalence of CR bacteria among Ukrainian patients (10/103; 9.7%; 95% CI: 4.8–17.1), all of these caused by carbapenemase genes, which is in agreement with data from the ECDC for Ukraine and other countries of eastern Europe [9]. In context with ECDC data on invasive isolates, 38.0% of *A. baumannii* and 10.0% of *K. pneumoniae* have been found to be CR isolates in Europe (excluding the United Kingdom) [9]. For the Ukraine, however, almost 77% of *A. baumannii* and almost 84% of *K. pneumoniae* in the tested invasive materials have been found to be CR isolates [9]. The number of patients positive for CR bacteria was almost 12-fold higher among Ukrainian than among non-Ukrainian (13/1,645; 0.79%; 95% CI: 0.4–1.3; p ≤ 0.01) patients tested on the day of admittance to the UHF within the investigated 4-month period. All Ukrainian patients positive for CR bacteria had either war-related injuries or a history of medical pre-treatment in Ukraine.

Our study revealed epidemiological differences between Ukrainian patients (prevalence of CR bacteria: 9.7%) and refugees from the Middle East who arrived at the UHF in 2015 and 2016 with a significantly lower but still elevated (0.9 and 2.1%, respectively) prevalence of CR bacteria [14,15]; therefore, adapted infection control measures for war refugees in hospitals seem necessary to prevent CR bacteria transmission. Of note, one third of the patients in our study either had war-related injuries (6/103) or were admitted to the Department for Paediatrics (29/103). Furthermore, four of 10 patients tested positive for CR bacteria were younger than 18 years. These findings indicate the need for adequate infection control measures in hospitals for these paediatric patients.

All Ukrainian patients positive for CR bacteria had either war-related injuries or a history of medical pre-treatment in Ukraine (as far as documented in the patient data file). When excluding these patients with war-related injuries or medical pre-treatment in Ukraine, the prevalence of MDRGN pathogens amounted to 5.3% (4/76; 95% CI: 1.5–12.9). This is not significantly different from the prevalence of MDRGN and CR bacteria in adult German patients admitted to an intensive care unit at the UHF (7.9%; 95% CI: 5.6–10.6) [15].

Interestingly, no MRSA was found in any of the Ukrainian patients, whereas the MRSA prevalence in refugees from the Middle East and German patients admitted to the UHF has formerly been shown to range around 5.0% and 1.0%, respectively [14,15]. This might indicate that patients arriving from Ukraine do not seem to be at elevated risk to carry MRSA. However, it must be stated that MRSA detection was done on chromogenic MRSA agar without additional enrichment cultures according with national recommendations [28] and that the MRSA prevalence in the study population might be in fact higher. Moreover, the absence of MRSA in our cohort of Ukrainian patients might also be influenced by the small size of that group. Use of antibiotics in relation to the time of screening might additionally have influenced MRSA prevalence in Ukrainian patients admitted to the UHF. However, information on antibiotic pre-treatment was not available due to language barriers.

Genetic characterisation of the MDRGN bacteria (n = 29) revealed nine different carbapenemase genes among the CR isolates, with *bla*_NDM‑1_ detected most frequently (n = 10). When excluding the two pairs with phylogenetically identical bacteria (related patients A and B; patients E and H with unclear linkage), no epidemiological linkage appeared between the remaining 25 isolates. Therefore, it is highly likely that spread of these resistance traits was the result of numerous underlying and independent events instead of a clonal spread. While it is obvious that during emergency and disaster medicine – particularly in war-related circumstances – infection control standards might not be fully achievable, it is of high importance to prevent further spread of these resistance traits within European hospitals.

Another remarkable finding of our study is the large proportion of CR *K. pneumoniae* positive for hypervirulence markers (hvKp; 5/6; see Supplementary Table S2). Such hvKp are capable of causing severe infections in otherwise healthy individuals. However, hvKp are usually less resistant and not common in healthcare settings [25]. Through exchange of resistance or hypervirulence plasmids, CR hvKp may develop into a novel threat for patients. Outside the Asian Pacific Rim, these pathogens have so far rarely been described, e.g. from Ireland [25,29]. This calls for timely and balanced anti-infective strategies in hospitals to guarantee best infection prevention for all patients, independent of their origin. With regard to the indication for in-hospital isolation measures, the German Infection Prevention Commission (KRINKO) at the Robert Koch Institute in Berlin, Germany, recommends isolation for patients who tested positive for e.g. CR bacteria [30]. Furthermore, isolation is recommended for patients who tested positive for MDRGN when admitted to e.g. intensive care units, intermediate care units or other high-risk units such as neonatology or haemato-oncological wards. Taking these criteria into account, isolation would have applied to 14 of the 103 (13.6%; 95% CI: 7.6–21.8) Ukrainian patients in our study group, in contrast to a slightly smaller amount of 12.1% resident patients, as previously shown [14].

Since we are presenting early data on the epidemiological situation on Ukraine patients admitted to a German university hospital, our findings rest on a small patient group which might have resulted in a limited scope for interpretation, e.g. because information on antibiotic pre-treatment was not available, in particular in relation to the time of screening. Nevertheless, preventing the spread of these isolates (as well as plasmids harbouring the respective resistance determinants) in hospitals is a central task to preserve antibiotic treatment options for critically ill patients. 

While epidemiological success of these MDRGN strains in hospitals is hard to predict, our study underlines the need for recent infection control strategies to cover the elevated prevalence of MDRGN pathogens in patients from Ukraine. Based on our findings, the UHF established the following infection prevention and control procedure: Screening and pre-emptive isolation of Ukrainian patients on the day of admittance is performed if (i) the patient is living in a refugee accommodation and/or (ii) presents war-related wounds and/or (iii) reports medical pre-treatment in a hospital in Ukraine. Ukrainian patients meeting (i) are released from isolation as soon as negative screening results for MDRGN bacteria (or MRSA) are available. Ukrainian patients meeting (ii) or (iii), however, remain isolated during their entire stay at the UHF. By adhering to this infection control strategy, at UHF no transmission of MDRGN to other patients has been observed until today.

## Conclusion

This early study addressed the prevalence and the molecular epidemiology of MDRGN and CR bacteria in Ukrainian patients at admittance to a hospital in Germany. Prevalence of MDRGN and CR bacteria is high in paediatric Ukrainian patients and those with war-related injuries or hospital pre-treatment in Ukraine. The occurrence of hypervirulent carbapenem-resistant *K. pneumoniae* isolates is a public health concern.

## Supplementary Data

110000 Supplementary_Figure

## Supplementary Data

59000 Supplementary_Tables
