# Supplementary material for: Molecular surveillance of multidrug-resistant Gram-negative bacteria in Ukrainian patients, Germany, March to June 2022
Source: Euro Surveill. 2023 Jan 5;28(1):2200850. doi: 10.2807/1560-7917.ES.2023.28.1.2200850 (PMC9817211; doi:10.2807/1560-7917.ES.2023.28.1.2200850)
Supplement: Supplementary_Figure [file 22-00850_REINHEIMER_SupplementaryFigure.pdf]

This supplementary material is hosted by Eurosurveillance as supporting information alongside the article "Molecular surveillance of multidrug-resistant gram-negative bacteria in Ukrainian patients", on behalf of the authors, who remain responsible for the accuracy and appropriateness of the content. The same standards for ethics, copyright, attributions and permissions as for the article apply. Supplements are not edited by Eurosurveillance and the journal is not responsible for the maintenance of any links or email addresses provided therein.

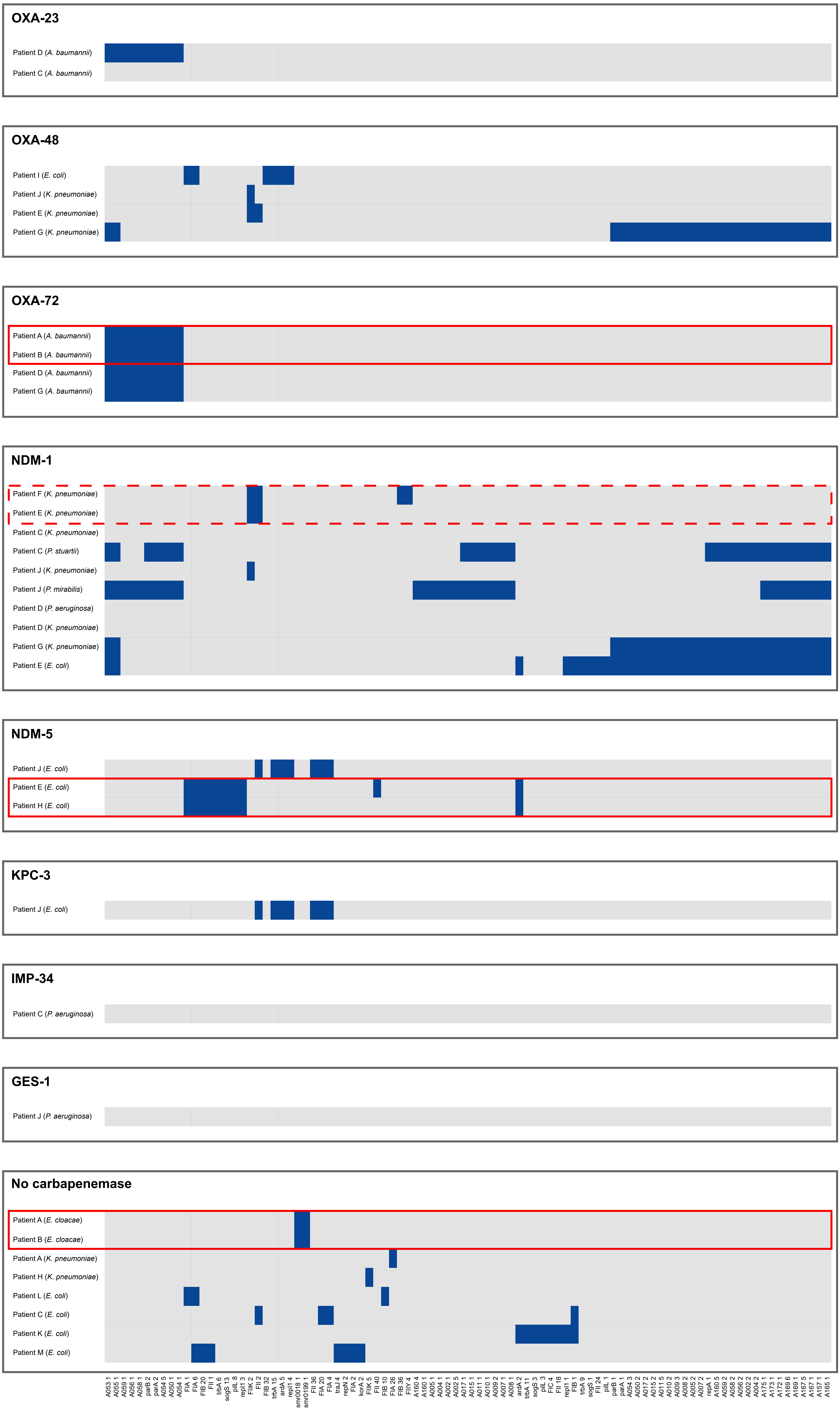

Figure S1. Analysis of plasmid allele types found in the genome of the 29 sequenced MDGRN and CR isolates of this study. Blue boxes indicate perfect matches for the respective allele type. Isolates are ordered by detected carbapenemase genes, therefore isolates with multiple carbapenemases appear multiple times. The three isolate pairs with close phylogenetic relatedness (<20 SNVs) in core genome analysis are highlighted by red boxes with a continuous line and the fourth potentially related pair (~50 SNVs) is marked by a red box with a broken line.
